# Computer-Assisted Cognitive Remediation in Schizophrenia: Efficacy of an Individualized *vs*. Generic Exercise Plan

**DOI:** 10.3389/fpsyt.2020.555052

**Published:** 2020-09-16

**Authors:** Magdalena Bossert, Celina Westermann, Thomas M. Schilling, Matthias Weisbrod, Daniela Roesch-Ely, Steffen Aschenbrenner

**Affiliations:** ^1^ Department of Clinical Psychology and Neuropsychology, SRH Klinikum Karlsbad-Langensteinbach, Karlsbad, Germany; ^2^ Department of Psychiatry und Psychotherapy, SRH Klinikum Karlsbad-Langensteinbach, Karlsbad, Germany; ^3^ Department of General Psychiatry, Center of Psychosocial Medicine, Heidelberg University, Heidelberg, Germany; ^4^ Centre for Psychosocial Medicine, Department of General Adult Psychiatry, Division Neurocognition, University of Heidelberg, Heidelberg, Germany

**Keywords:** cognitive remediation, drill and practice, schizophrenia, exercise plan, neuropsychology

## Abstract

Computer-assisted cognitive remediation (CACR) is an economical, adjustable, and effective treatment for individuals with schizophrenia. The current randomized controlled study examined whether an individualized or generic exercise plan in CACR is superior in patients with multiple cognitive deficits compared to treatment-as-usual (TAU). Fifty-nine inpatients diagnosed with schizophrenia were randomly assigned to 1) TAU, 2) TAU plus an individualized exercise plan in CACR, or 3) TAU plus a generic exercise plan in CACR. Neuropsychological performance, psychopathology, and functional outcome were assessed at baseline and post-treatment. The results show a medium to large training effect for all neuropsychological performance measures. Contrary to our expectations the neuropsychological improvement over time did not differ between groups. Self-reported depression, global level of functioning, and activity and participation functioning showed a significant improvement from baseline to post-treatment. However no further group, time, or interaction effects for other psychopathology and functional outcome could be demonstrated. Possible implications for clinical use of CACR and future studies are discussed.

## Introduction

The Cognitive Remediation Expert Working Group defines cognitive remediation as a “behavioral training intervention targeting cognitive deficits (attention, memory, executive function, social cognition, or metacognition), using scientific principles of learning, with the ultimate goal of improving functional outcomes. Its effectiveness is enhanced when provided in a context (formal or informal) that provides support and opportunity for extending to everyday functioning” (1). Computer-assisted cognitive remediation (CACR) has the advantage of being economic by lowering the global health care costs (2) and enhancing productivity outcomes (3). Furthermore CACR can be adaptive, repeated infinitely, provide incentives and be tailored to specific cognitive domains (4, 5).

So far, two major meta-analyses have examined the efficacy of CACR (5, 6). Prikken, Konings (6) meta-analysis included 24 studies using CACR for improving cognitive functioning in schizophrenia. They report significant small to medium treatment effects for attention, working memory, positive symptoms, and depressive symptoms. However they questioned the generalization of treatment effects due to the absence of further significant effects on other neuropsychological measures as well as functional outcome and social cognition.

The main conclusion of the second major meta-analysis (5) is that the effects of CACR on cognitive domains do not differ, regardless whether the cognitive domain is specifically targeted by CACR or not. However, the authors drew this conclusion indirectly, despite the fact that so far no study was specifically designed to test this hypothesis. Indeed, the majority of studies included in the meta-analysis seems unsuited to answer the question whether a generalized or individualized exercise plan is more efficacious: first, most studies only report pre- and post-treatment effects of those cognitive domains that where specifically intended to treat and did not test for generalization effects. Moreover, although the training software is not specifically mentioned in the meta-analysis, the original studies mainly used old-fashioned programs that lack the motivational, incentive, and multimedia-based opportunities current CACR can offer. Thus, these findings might not extend to current, state-of-the art CACR programs. In German speaking countries mainly CogPack, RehaCom, and CogniPlus are currently used as well-evaluated CACR programs (7). In the present study we used CogniPlus as CACR program, whose efficacy has been shown (e.g., (8–10)) and is recommended by the German Association of Neuropsychology (GNP—Gesellschaft für Neuropsychologie). The program is based on a function-specific intervention approach, with a motivating design and automatic adjustment of task difficulty. Thus the current study fulfills the requirements for modern CACR programs. Recent studies focus even more on motivating aspects of psychosocial rehabilitation by considering video games as possible treatment strategy (11).

Another limiting factor to the meta-analysis of Grynszpan, Perbal (5) is that majority of the 16 included studies had a low training frequency, with seven out of 16 only providing two training sessions a week and 9 out of 16 only providing 16 or less training sessions in total. One of the exceptions is the study of Kurtz, Seltzer (12). They compared the effects of an extensive CACR program on neuropsychological functions with a medium of 67 h of training over a 12 month period with an active control condition receiving computer skill training (71 h of training on average). Their findings suggest that nonspecific computer based stimulation in the control condition had a salutary effect on neurocognitive functions like working memory, reasoning/executive-function, verbal and spatial episodic memory, and processing speed as well. Only for working memory a significant interaction effect could be detected indicated a greater pre-post-improvement of the CACR group. The authors concluded that different types of sustained and goal-directed cognitive activity might improve neurocognitive skills and that the control condition plays a crucial role in study outcome. They missed to take other outcome measures such as psychopathology or social functioning into consideration.

The low training frequency of most previous studies is of special relevance, since intensity and frequency of CACR seem important for specific treatment effects (13, 14). Possibly, low dosed CACR might early on lead to general cognitive improvements, whereas specific cognitive treatment effects require high frequent, intense CACR, and appear only after a sufficient number of training sessions.

To our knowledge no study has yet explicitly investigated the potentially influence of a generic versus individualized exercise plan on neuropsychological measures, psychopathology, and functional outcome. The current study was therefore designed to address the question, whether the outcome and efficacy of CACR in patients with schizophrenia with multiple cognitive impairments differ depending on the exercise plan. We conducted a three-group randomized, controlled, repeated measure study where patients received 1) TAU, 2) trained their individual three main cognitive impairments (I-CACR), or 3) trained all six measured cognitive domains (G-CACR) 4 days in a week. Following current recommendation on CACR for schizophrenia intense drill and practice CACR was combined with a weekly transfer session including problem solving and transfer to real world functioning (14). Neuropsychological functioning as well as psychopathology and functional outcome were assessed both at baseline and after 5 weeks of high frequent CACR. We assumed that an individualized exercise plan (I-CACR) would lead to greater improvements in individually impaired cognitive functions due to restorative, intense drill, and practice based recovery. On the other hand we expected that a generalized exercise (G-CACR) plan might lead to more widespread effects in psychopathology and functional outcome. Further, we expected both CACR-groups to outperform the treatment-as-usual group (TAU) that received standard medical treatment but no explicit cognitive training.

## Method

### Design

**Figure 1:**
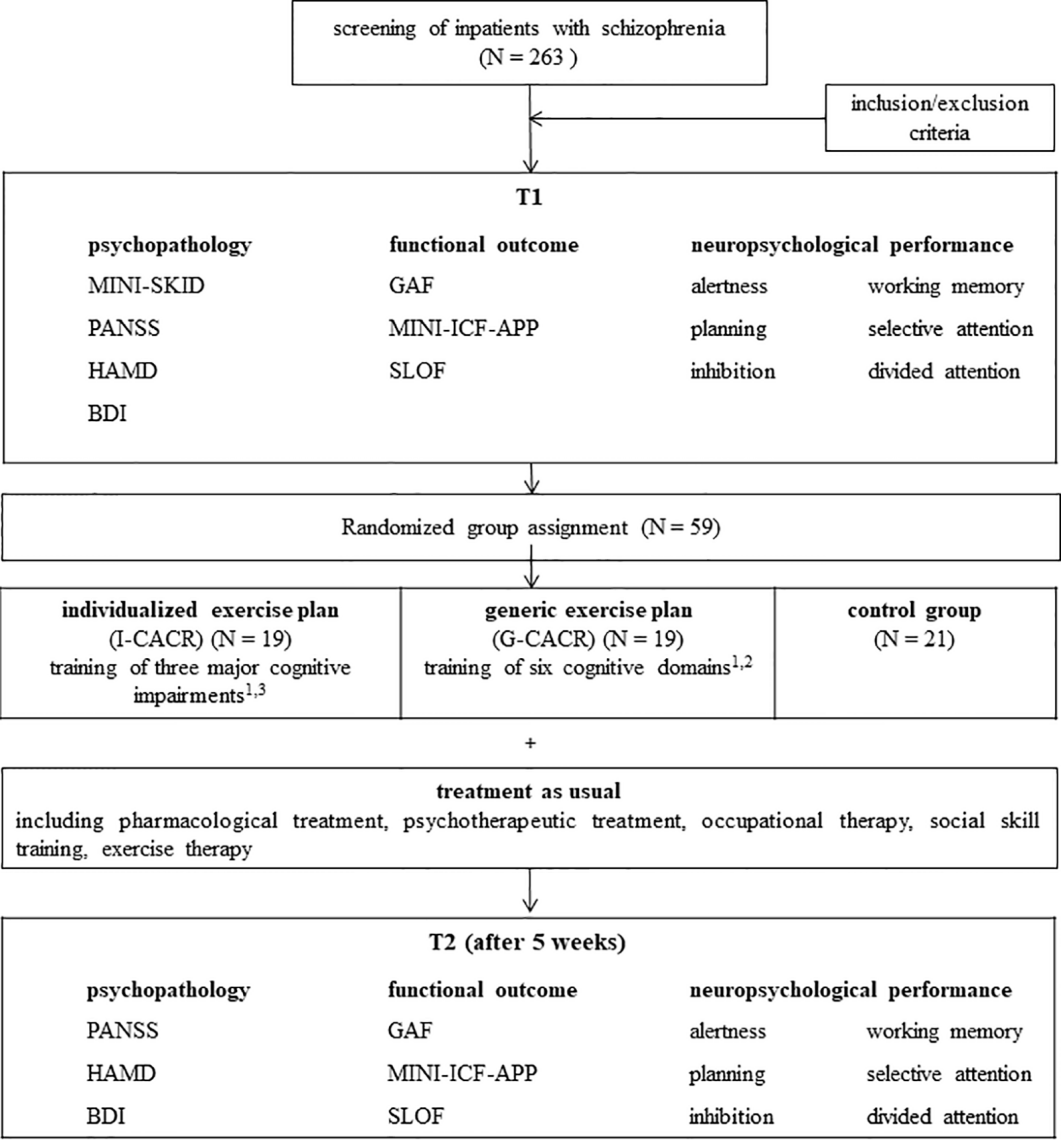
Schedule of the study. ^1^ Four sessions per week, additional one transfer session (individual benefits and motivation, problem solving strategies, transfer to real world functioning). ^2^ Alertness, divided attention, selective attention, planning, working memory, inhibition. ^3^ Three main deficits out of the six subdomains, depending on individual results of neuropsychological performance at T1.

We conducted a randomized controlled pre-post-intervention study (see **Figure 1**). During admission routine all inpatients diagnosed with schizophrenia not suffering from acute psychotic symptoms were screened at the section for psychiatry and psychotherapy of the SRH medical center Karlsbad-Langensteinbach. Inclusion criteria were age between 18 and 55 years, IQ > 80 in the Multiple Choice Vocabulary Intelligence Test [MWT-B (15)] and cognitive deficits (defined as percentile rank <16 in the German speaking norm sample) in at least three out of six cognitive subdomains. Exclusion criteria were neurological diseases, current substance abuse as well as acute positive symptoms (PANSS: positive scale ratings > 5). At baseline sociodemographic information were collected through an interview specially designed for this study. All participants were diagnosed and rated by a trained clinical therapist using the short version of the structured clinical interview for Diagnostic and Statistical Manual of Mental Disorders (DSM)-IV (MINI-SKID; German version). In addition baseline assessment included psychopathology, neuropsychological performance, and functional outcome measures, which are described in more detail below. After baseline assessment subjects were randomly assigned to one of three treatment condition (TAU, I-CACR, or G-CACR). After completing 5 weeks of CACR or TAU all subjects were reassessed non-blinded by a trained clinical therapist. To exclude potential rater bias, psychopathology and functional outcome were rated under clear guidelines and neuropsychological functions were assessed with a standardized, computer-based battery.

### Subjects

Fifty-nine chronically ill patients met inclusion criteria, gave written informed consent, and participated in the study. The total sample consisted of 43 males and 16 females, aged between 19 and 50 years (M = 30.2, SD = 8.3). Most of the patients were diagnosed with paranoid schizophrenia [10th Revision of the International Statistical Classification of Diseases and Related Health Problems (ICD-10): F20.0] (88.7%), other diagnoses included hebephrenic schizophrenia (ICD-10: F20.1) (3.2%), catatonic schizophrenia (ICD-10: F20.2) (1.6%), or not otherwise specified schizophrenia (ICD-10: F20.9= (1.6%). 18.6% (N = 11) had one or more comorbid ICD-10 diagnoses including psychological and behavioral disorders resulting from psychotropic substances (17%), neurotic, stress-related, and somatoform disorders (3%) as well as affective disorders, personality and behavioral disorders and behavioral and emotional disorders with onset usually occurring in childhood and adolescence (each 1.6%). The mean drug dosage was 555 mg/d (SD=413) (mean chlorpromazine equivalent dose). Fifty percent of the subjects received further medication [antidepressants (29%), anticholinergics (12%), thyroidhormones (7%), anticonvulsive drugs (6%)]. Cognitive deficits appeared with a frequency of 86% in divided attention, 85% in response inhibition, 81% in selective attention, 66% in working memory, 59% in alertness, and 44% in planning. The majority of the subjects were unemployed (71%). The remaining were employee (3%), apprentice/student (7%), pensioner (5%), and others—like voluntary worker or trainee (14%). The subjects had rather high education level with 37% completing A-level, 32% high school diploma, 27% secondary school, and 3% without graduation. The medium duration of disorder was 64 month (SD=61.00), with a medium onset at the age of 25 (SD = 8.70), and an average of 3.15 (SD= 2.08) inpatient stays. The study was approved by the Ethical Committee of the University of Heidelberg and was in accordance with the latest revision of the declaration of Helsinki. It has been registered with DRKS (Deutschen Registers Klinischer Studien: Registration trial DRKS00021628) and is searchable *via* the WHO meta-registry (http://apps.who.int/trialsearch/).

### Instruments

Six neuropsychological domains were tested *via* the Vienna test system (Schuhfried, Mödling, Austria) (16): 1) alertness, 2) divided attention, 3) selective attention, 4) working memory, 5) planning, 6) response inhibition. The program is very commonly used and has standardized norms for German speaking countries. It directly links the neuropsychological testing to the CACR with CogniPlus and was therefore chosen over the MATRICS Consensus Cognitive Battery (17, 18). Furthermore the MATRICS Consensus Cognitive Battery has no normative data for German-speaking countries and the mere translation of the different tests might lead to potential cultural influences. Due to the main research question a German CACR software was required, with clearly defined neuropsychological constructs. Within CogniPlus (19) six function-specific interventions were used for the study: ALERT (alertness), SELECT (selective attention), DIVID (divided attention), HIBIT-R (inhibition), PLAND (planning), NBACK (working memory). A detailed description of neuropsychological assessment and training is available on the Schuhfried website (https://www.schuhfried.com/).

Clinical assessment included the structured clinical interview for DSM-IV (MINI-SKID; German version), Positive and Negative Syndrome Scale (PANSS), and 24-item Hamilton Rating scale for depression (HAM-D) and a sociodemographic interview. Functional outcome measures were the Global Assessment of Functioning Scale (GAF), the Specific Level of Functioning Scale (20), and the Short Rating of International Classification of functioning (21) (**Table 1**).

**Table 1:** Overview of Assessment.

Dimension	Description
**Psychopathology and demographics**	
PANSS	Positive and Negative Syndrome Scale (22)
HAMD	Hamilton Rating Scale for Depression (23)
M.I.N.I.-SKID	Clinical interview based on DSM-IV (only pre-testing) (24)
BDI	Beck depression inventory (25)
MWT-B	Multiple Choice Vocabulary Intelligence Test (*estimation of intelligence*) (only pre-testing) (26)
**Functional outcome**	
GAF	Global Assessment of Functioning [Diagnostic and Statistical Manual of Mental Disorders (DSM-IV)]
MINI-ICF-APP	Mini-International Classification of Functioning-Rating for activity and participation impairments in mental illnesses (27)
SLOF	Specific level of functioning
**Neuropsychological performance**	
Attention Executive functions	Divided attention: Vienna Test System WAFG Selective attention: Vienna Test System WAFS Alertness: Vienna Test System WAFA Planning: Vienna Test System Tower of London TOL Inhibition: Vienna Test System Go-Nogo INHIB Verbal working memory: Vienna Test System N-back verbal NBV

### Intervention

After baseline assessment the patients were randomly assigned to one of the three treatment conditions. Core techniques for cognitive remediation for schizophrenia were used during the study (14). The results of the cognitive pre-testing and the associated effects of cognitive deficits on mental health and everyday functioning were communicated and explained to the subjects during an individual therapy session before starting CACR. The control group received treatment-as-usual (TAU) including pharmacological and psychotherapeutic treatment, occupational therapy, and social skill training among others. The two intervention groups additionally received CACR. The training session was conducted as a group CACR with up to six participants. CACR was supervised and instructed by clinical psychologists (university degree), who had further knowledge in the field of neuropsychology. All therapists obtained weekly supervision by an experienced neuropsychologist. CACR was conducted as an intense training including four 50-min-sessions per week for 5 weeks with mere computerized cognitive training. Before starting CACR an individual introduction on how to navigate the computer program and an explanation of every training program was given. CogniPlus automatically adjusts task difficulty to the individual progress and allows tracking of performance parameters such as speed and accuracy. After each training unit the therapist gave an individual feedback including performance parameters. Whereas patients of the G-CACR group trained all six subdomains, patients of the I-CACR group trained their three main individual deficits objectified in the baseline assessment (74% divided attention, 68% response inhibition, 58% selective attention, 42% working memory, 36% alertness, 21% planning). During an additional 50-min group session per week background information of CACR was provided. During these sessions the individual advantages of cognitive improvements on everyday functioning were discussed, hereby promoting motivation and goals, addressing barriers, and exchanging strategies.

### Data Preprocessing

After data screening for outliers and invalid data entry the raw values of baseline and post-treatment assessments of each neuropsychological test were jointly standardized (t-scored) according to the whole sample separately for each performance measure of the test (e.g., divided attention: reaction time, standard deviation of reaction times, omissions, false alarms; each performance measures was individually standardized to the sample). Standardized data was then checked for outliers, which were truncated to ±2 SD from the mean of the whole sample. Next, for each neuropsychological test one performance index per person was calculated by averaging the different standardized performance measures resulting in one performance index per neuropsychological test per person (e.g., performance index of divided attention: average of the standardized data of reaction time, standard deviation of reaction times, omissions, false alarms). Performance indices were used for all further analysis.

### Statistical Analyses

All statistical analyses were carried out using IBM SPSS Statistics Version 25 for Windows. A critical alpha level of α = 0.05 (two-tailed) was deemed significant. For categorical data relative frequencies were used, comparisons were carried out using a χ2-test. Sociodemographic as well as psychopathological data and data concerning the functional outcome were displayed using means and standard deviations. Comparisons of means between the three different treatment groups were performed parametrically using univariate ANOVA. For each dependent variable separate 2 TIME (T1: baseline; T2: post-treatment) x 3 GROUP (TAU; I-CACR; G-CACR) mixed-model ANOVAs were calculated.

## Results

### Training Attendance

Of the total of 25 training sessions (including one transfer session per week) mean attendance of the I-CACR group was *M* = 22.95, *SD* = 3.43, and of the G-CACR group was *M* = 22.26, *SD* = 3.17. Groups did not differ in their mean attendance rate (*t* (26) < 1, *p* = 0.53).

### Psychopathology and Demographics

There were no differences between the three groups at baseline neither in PANSS, Hamilton Depression Rating Scale (HAMD), or the Beck Depression Inventory (BDI)-ratings nor in age, sex, onset of disease, duration of disease, or education (see **Table 2**). Psychopathological ratings in PANSS and HAMD did not change from pre- to post-intervention (PANSS: TIME [*F* (1, 56) < 1, *p* = .69, TIME x GROUP [*F* (2, 56) < 1, *p* = .70]; HAMD: TIME [*F* (1, 56) = 2.48, *p* = .121; TIME x GROUP: F (2, 56) < 1, *p* = .88]. For BDI-scores a main effect of TIME [*F* (1, 52) = 22.82, *p* <.001, *η_p_²*= .31] was observed, indicating a general improvement (i.e., lowering in scores) in self-reported depression. However, no interaction TIME x GROUP [*F* (2, 51) = 1.04, *p* = .360] was found. Hence, except for a general improvement in self-reported depression in all groups psychopathological symptoms did not change pre- to post-intervention.

**Table 2:** Baseline assessment of demographics, psychopathology, and functional outcome.

	I-CACR *n* = 19		G-CACR *n* = 19		TAU *n* = 21		*F*	*p*
	Mean	*SD*	Mean	*SD*	Mean	*SD*		
Age	32.37	8.71	28.68	9.43	29.67	6.65	1.01 (2, 56)	.37
Sex *n* (%)								
Male	13 (68)		14 (74)		16 (76)			
Female	6 (32)		5 (26)		5 (24)			.86¹
Onset of disease (age)	25.58	9.48	24.95	9.97	24.57	6.98	0.06 (2, 56)	.94
Duration of disease (months)	83.05	75.81	48.89	52.86	59.71	50.23	1.59 (2, 65)	.21
Education (years)	14.63	4.78	12.74	2.08	14.62	3.72	1.69 (2, 56)	.20
PANSS								
Positive scale	9.53	2.85	9.63	3.72	9.95	3.11	0.10 (2, 56)	.91
Negative scale	13.74	6.67	14.21	5.72	15.05	5.81	0.24 (2, 56)	.79
Global scale	24.47	6.28	22.68	5.32	25.00	5.17	0.92 (2, 56)	.40
HAM-D	8.47	4.789	6.00	3.830	7.67	4.531	1.56 (2, 52)	.22
BDI	14.88	8.162	12.67	13.750	15.74	9.048	0.42 (2, 52)	.66
MWT-B	28.17	4.93	25.72	4.03	24.81	4.46	2.84 (2, 54)	.07
GAF	57.89	8.219	58.42	6.678	57.62	8.605	0.05 (2, 56)	.95
MINI-ICF-APP	24.28	9.821	23.56	8.368	27.47	8.695	0.56 (2, 56)	.58
SLOF	97.95	9.986	97.68	9.399	90.81	12.352	2.89 (2, 56)	.06

SD, standard deviations; F, ANOVA (including degrees of freedom). ¹By chi-square test.

### Functional Outcome

Again, there were no differences between the three groups at baseline in summed International Classification of Functioning, Disability and Health (ICF), summed Specific Levels of Functioning Scale (SLOF), and GAF ratings (see **Table 2**). In GAF a main effect of TIME [F (1, 52) = 20.42, *p* <.001, *η_p_²* = .27], reflecting a general increase in the global level of functioning in all patients, but not TIME x GROUP [F (2, 56) < 1, *p* = .95) interaction was observed. Likewise, analyses on the summed up MINI-ICF-APP score showed an effect of TIME [F (1, 52) = 29.21, *p* <.001, *η_p_²* = .36], indicating a general improvement in functioning in all groups, but no interaction TIME x GROUP [*F* (2, 52) < 1, *p* = .97]. For the summed up SLOF ratings neither a main effect of TIME [*F* (1, 56) < 1, *p* = .54] nor an interaction TIME x GROUP [F (2, 56) < 1, *p* = .78] was observed.

### Neuropsychological Performance

No group differences pre-intervention were detected in any of the neuropsychological performance indices (see **Table 3** for simple main effects “group at T1” of the six neuropsychological performance indices). Mixed-model ANOVAs of all neuropsychological performance indices yielded main effects of TIME [all *F* (1, 2) > 4.5, all *p* <.05], indicating that participants of all groups improved in their neuropsychological performance from the pre- to the post-intervention measurement time point. The effect size reached from moderate to large (*η_p_²* = 0.08 to 0.29). Nevertheless, neither a main effect for GROUP [all *F* (2, 56) < 2.0, all *p* >.05], nor an interaction TIME x GROUP [all *F* (2, 56) < 2.2, all *p* >.05] was found in any neuropsychological index. Thus, improvement over time did not depend on group membership. See **Table 3** for exact data.

**Table 3:** Group comparison.

Neuropsychological index/test	Group	T1		T2		Effect	*F*	*P*	*ηp2*
		Group M	Group SD	Group M	Group SD				
Divided attention (WTS-WAFG)						GROUP at T1	0.41	0.67	0.01
	I-CACR	50.39	5.30	48.38	4.71	TIME	6.64	0.01	0.11
	G-CACR	51.81	7.24	49.92	8.17	GROUP	0.49	0.62	0.02
	TAU	50.07	6.53	48.14	7.27	TIME x GROUP	<0.01	1.00	0.00
Selective attention (WTS-WAFS)						GROUP at T1	0.49	0.62	0.02
	I-CACR	52.60	7.21	48.82	5.76	TIME	21.02	<0.01	0.27
	G-CACR	52.08	7.19	47.62	6.58	GROUP	0.45	0.64	0.02
	TAU	50.40	7.72	47.48	5.16	TIME x GROUP	0.31	0.74	0.01
Alertness (WTS-WAFA)						GROUP at T1	0.48	0.62	0.02
	I-CACR	51.06	8.59	48.66	6.99	TIME	15.14	<0.01	0.21
	G-CACR	52.08	7.40	48.06	5.81	GROUP	0.06	0.94	0.00
	TAU	49.82	5.72	48.97	5.99	TIME x GROUP	2.18	0.12	0.07
Planning (WTS-TOL)						GROUP at T1	0.62	0.54	0.02
	I-CACR	46.17	10.37	53.14	11.12	TIME	16.81	<0.01	0.23
	G-CACR	47.38	9.27	49.80	10.02	GROUP	0.52	0.60	0.02
	TAU	49.39	8.11	53.37	9.06	TIME x GROUP	1.46	0.24	0.05
Response inhibition (WTS-INHIB)						GROUP at T1	2.44	0.10	0.08
	I-CACR	52.04	6.76	49.67	6.81	TIME	4.58	0.04	0.08
	G-CACR	52.02	7.49	49.69	8.05	GROUP	1.47	0.24	0.05
	TAU	48.38	3.46	47.88	6.51	TIME x GROUP	0.60	0.55	0.02
Verbal working memory (WTS-NBV)						GROUP at T1	0.65	0.52	0.02
	I-CACR	51.57	5.85	49.14	4.22	TIME	5.74	0.02	0.09
	G-CACR	49.62	5.43	48.68	6.71	GROUP	0.33	0.72	0.01
	TAU	51.90	8.33	48.93	7.07	TIME x GROUP	0.47	0.62	0.02

t-scored results of neuropsychological performance indices separated by index and group and main and interaction effects. Note, lower t-scores indicate better performance except for planning (WTS-TOL) where higher values indicate better performance. All neuropsychological testing was carried out using the Wiener Test System of Schuhfried, Vienna, Austria (WTS), the specific neuropsychological test is stated along with the neuropsychological index. Annotation: I-CACR, individualized CACR; G-CACR, generic CACR; TAU, treatment as usual. GROUP at T1, simple main effect of group pre-treatment to assess pre-existing baseline-differences; TIME, GROUP, TIME x GROUP, main and interaction effects of the 2 TIME (T1: pre-treatment; T2: post-treatment) x 3 GROUP (TAU; I-CACR; G-CACR) mixed-model ANOVAs.

## Discussion

The efficacy of CACR in the treatment of people with schizophrenia is extensively displayed in literature and CACR has the advantage of being motivating, adjustable, and economical (3, 14). The meta-analysis of Grynszpan, Perbal (5) concludes that treatment effects on cognitive domains that were explicitly targeted by CACR do not differ from those domains that were not. However, to the best of our knowledge no study has explicitly adjusted the CACR exercise plan to either individual impairments or to generic domains and investigates the impact on neuropsychological performance, psychopathology, and global functioning. Therefore, we conducted a randomized controlled study, where patients received 1) TAU, 2) trained their individual three main cognitive impairments (I-CACR), or 3) trained all six measured cognitive domains (G-CACR). We used recommended CACR techniques for schizophrenia and assessed neuropsychological performance, psychopathology, as well as functional outcome at baseline and post-treatment. The results showed a medium to large time effect for all neuropsychological domains (divided attention, selective attention, alertness, planning, working memory, inhibition), self-rated-depression, global level of functioning, and activity and participation functioning but no group or interaction effect, indicating that neither group was superior or that the change over time differed between groups.

Contrary to our expectations we neither observed stronger improvements of the training groups *vs*. TAU-group nor an advantage of the I-CACR *vs*. G-CACR. The homogeneity in improvement between the two treatment groups is in line with the meta-analysis of Grynszpan, Perbal (5) and strongly supports their conclusion that an individualized exercise plan is not more efficacious than a generic one. Thus, a “one size-fits-all” approach seems to be as effective as an individually tailored training plan. However, unexpectedly, the training groups did not outperform the TAU-group.

With medium to large time effects on neuropsychological performance we must assume a strong TAU. A meta-analytic review of non-specific effects in randomized controlled trials of Cognitive Remediation for schizophrenia showed that control groups in 32 CR trials showed small effect size changes (Cohen’s d = 0.12 ± 0.16) in cognitive test performance (28), indeed much smaller than the ones observed in the current study. They recommend a greater attention to change in control groups to detect cognitive remediation effects. The clinic in which the data of the current study was collected is specialized in occupational rehabilitation, therefore several components of TAU such as occupational therapy, exercise therapy, social skill training, or psychotherapy might lead to cognitive improvements as well. Even though the needed sample size was calculated in advance using G-power assuming a moderate effect (1-β = 0.9; d = 0.5, n = 18 per group), in hindsight the sample size might have been too small to detect possible smaller additional treatment effects of CACR.

Other previous studies comparing CACR with active control groups also failed to show group differences (4, 29, 30). Linke, Jankowski (30) compared CACR in inpatients with schizophrenia with an active control group over a 6 week period. Both groups improved similarly in cognitive function and psychopathological symptoms. Only the reduction of negative symptoms in CACR was more efficient. For early stages of schizophrenia Garcia-Fernandez, Cabot-Ivorra (4) showed no significant group differences comparing 24 1-h-sessions of REHACOM with an active control group for clinical features, cognition, and functioning.

Understanding the heterogeneity of responses to CACR helps to identify factors that improve the effectiveness. The current meta-analysis of Prikken, Konings (6) emphasis, that even though relevant effects of CACR on attention, working memory, positive symptoms, and depression in schizophrenia could be demonstrated, the inability of transferring treatment effects to functional outcome should stimulate further development of CACR. The current study showed no specific treatment effects by adjusting the exercise plan to individual impairments. Therefore, other underlying factors should be considered in future studies.

## Data Availability Statement

The raw data supporting the conclusions of this article will be made available by the authors, without undue reservation.

## Ethics Statement

The studies involving human participants were reviewed and approved by Ethical Committee of the University of Heidelberg. The patients/participants provided their written informed consent to participate in this study.

## Author Contributions

Authors SA, MW, DR-E, and CW designed the study. MB and CW organized data collection and literature search. MB, TS, and CW undertook the statistical analysis, and author MB wrote the first draft of the manuscript. All authors contributed to the article and approved the submitted version.

## Funding

The authors declare that this study received funding from Schuhfried GmbH. The funder provided the computerized test battery (CogBat^®^) and training program (CogniPlus^®^). The funder was not involved in the study design, collection, analysis, interpretation of data, the writing of this article or the decision to submit it for publication.

## Conflict of Interest

The authors declare that the research was conducted in the absence of any commercial or financial relationships that could be construed as a potential conflict of interest.
